# Collectanea Medica, Consisting of Anecdotes, Facts, Extracts, Illustrations, Queries, Suggestions, &c.

**Published:** 1818-09

**Authors:** 


### COLLECTANEA MEDICA,

CONSISTING OF

### ANECDOTES, FACTS, EXTRACTS, ILLUSTRATIONS, QUERIES, SUGGESTIONS, &c.

RELATING TO THE

*History or the Art of Medicine, and the Auxiliary Sciences.*

Quicquid agunt medici,
nostri farrago libelli.

### *Cases illustrating the beneficial Effects of Digitalis in Epilepsy.*

IN the second Number of the *American Medical Recorder*, two cases of Epilepsy are related, which were cured by digitalis. The first, a child two years of age, had been subject to epilepsy from the time it was two weeks old. It was in other respects in good health, and uncommonly fat. Five drops of the tincture of digitalis were ordered to be taken night and morning, and to be increased two drops per dose every six days.—The disease was removed within a fortnight. The other, a child four years old, had been subject to the complaint for two years: it had not suffered much from teething, and was not apparently affected with worms. A few days after taking the medicine the disease had disappeared.

The use of digitalis in epilepsy, when accompanied with vascular excitement, has been recommended by Dr. Currie, and some other medical practitioners of eminence; but we believe it has not been adopted to the extent so powerful a remedy appears to merit.

The same Journal contains an account of a case of mania *(delirium?)* from excessive drinking of spirituous liquors, cured by emetics. The patient, a man twenty-nine years of age, had been in a state of mental derangement during three or four days, when Dr. Eberle, of Philadelphia, saw him. An emetic was administered, which operated powerfully; he passed a tolerable night after it, and on the following days was much relieved. Another emetic was given, which perfectly restored him.

We transcribe from the same Journal the following case of *Phlegmasia dolens Puerperarum;* related by Dr. TALIAFERRO; considering every well-authenticated case of that serious disease worthy of attention; and, particularly so, those which have terminated favourably.

" In the month of February, 1818, I was called to see a woman who had, some twelve or fifteen days previous, been delivered of a child; and found, upon inquiry, that she was afflicted with what is termed a ' swelled leg.' She informed me that, about fortyeight hours previous to my being sent for, a pain and stiffness were felt in the groin, near the passage of the round ligament; shortly after which, the swelling commenced; and, upon examination, I was surprised to see the size of the limb, for it was at least double the ordinary size; it had a smooth shining appearance, and was very hot, though not red; the lochial discharge was not interrupted, nor was there much pain about the region of the womb. There were several lumps on different parts of the leg, which I supposed to be muscular contractions. The pulse was remarkably frequent, at least 140 in a minute; the skin dry; smart thirst; and, in fact, many marks indicative of considerable inflammatory excitement. I immediately drew about sixteen ounces of blood, gave a smart purge, and applied a large blister-plaster to the groin, extending up the abdomen. Next day my patient was no better; I, therefore, bled her again, ordered her to drink liberally of cream of tartar, and commenced bathing the limb with a solution of the acetate of lead. On the third day a mild cathartic was administered; the bathing with lead continued. By this time, the increased excitement was considerably reduced, but no abatement in the pain or swelling; on the contrary, the pain appeared to be greater, for she would frequently scream out aloud on her leg being moved. My patient expressed a great desire to have her leg bathed in bitter herbs: as this was a prescription of her own, and appeared simple, she was indulged in it for three or four days; but I believe it produced an injury, and was ordered to be discontinued. I now had recourse to opium, to allay irritation, which had been very considerable from the commencement. I commenced by giving one grain and a half at a dose, but found that quantity too small, and increased it to from two grains and a half to three at a dose, which was repeated as often as occasion might require, with the happiest effects; at the same time I commenced using the opium, I had the limb well rubbed with camphorated spirits three or four times every twenty-four hours; a mild cathartic was occasionally administered to prevent costiveness. This course was pursued for four or five weeks, when medicine became unnecessary; and, in about three months, she completely recovered."

---

*On the Temperature of the Summers which are adapted to give activity to the Infection or Seeds of the Yellow-Fever, in the City of Philadelphia.* (From the American Daily Advertiser.)

On the first day of August, 1809, there was published in the American Daily Advertiser, and, on the same day, or within a few days after, in most of the other daily papers of this city, an account which I had prepared of the state of Fahrenheit's thermometer, taken in the shade at 3 P.M. during seventeen summers, 1793 to 1809, both inclusive. I shall now republish that account, and subjoin a state of the same instrument from 1810 to 1817, so as to comprehend in all twenty-five summers, 1793 to 1817. The intent of this publication is to exhibit to public view the mean heat at 3 P. M. of each and every of those summer months, and to draw an inference from thence, that the yellow-fever, being a native of hot climates, cannot probably prevail to any alarming extent here in any season, except the mean heat of the thermometer at that hour, during the summer, and especially during the two whole months of June and July, shall be as high as seventy-nine degrees —if cooler, it will not spread, although some passengers and their clothing and bedding may arrive here, bringing the disease or the infection with them; but, in hotter seasons it has prevailed, and, probably, will again prevail more or less, and very much in proportion to the heat of the season.

This is not an inquiry prompted by idle curiosity, but an attempt to establish a knowledge very important in its consequences, not only to all our citizens concerned in naval or mercantile business, but to the whole population of this city and liberties; because, if well founded, as I believe it to be, it will serve as a rule to point out to every citizen when there is, or is not, danger to be apprehended; when it may be necessary, or not, to provide retreats in the country. If well understood and established to be a truth founded in experience, it may also tend to disembarrass the trade of this port, in some years, from detention and quarantines when they are useless.

By the following account of the mean heat at 3 P. M. of each month in the last twenty-five summers, it will appear evidently that the yellow-fever has never, within that period, prevailed here at all, or so as to occasion alarm, when the mean heat at that hour of all June and July had been lower than 79°, only a very little in 1802; and that, in every summer when it has been above 79°, it has prevailed more or less, and the mortality has been regulated very much by the heat being higher or lower. In 1793 and 1798, which were the hottest summers in all the twenty-five years, it prevailed most, and was attended by the most extreme mortality. In 1797, 1799, 1803, and 1805, when lower degrees of heat prevailed, the mortality was less. In all the other years, (except a small mortality in 1802,) when the mean heat of those two months was below 79° at the hour mentioned, we have had no alarm of yellow-fever.

I consider the two months of June and July as governing the summer season, insomuch that by the first day of August, in any year, we may be pretty certain whether we shall be afflicted with yellow-fever that year or not; so that if we find the mean heat of the thermometer at 3 P. M. placed properly in the shade, in a free current of air, at least twenty or thirty feet from any sunshine, and not exposed to the reflected heat of any building, to be below 79°, we may rest easy, and conclude that we are not likely to be visited with that scourge during the summer or autumn then passing over our heads.

In 1793, the mean heat of June and July at 3 P. M. was 82°; in 1798, it was 80° 6; both of which indicated the calamity that followed; but August, 1798, was so extremely hot, that it heightened the mortality, and made it nearly equal to what it was in 1793, when the two first summer months were hotter, but August not so hot as in 1798. The wetness or dryness of the summer may also have an effect, not yet well ascertained; it being remarkable, that in 1805, when the mean heat of all June and July was 79°, and August 81°7, the two months of July and August were so very dry, that perhaps not so much as one quarter of an inch in depth of rain fell till within three or four days of the end of the latter month, when it rained moderately: this rain appeared to be sufficient, coming after the preceding heat, to give activity to the dormant infection of the yellow-fever, which immediately afterwards broke out, more especially in Southwark, where it was very mortal in all September. The use of the Schuylkill water, which is said to be much purer than the old pump-water, may have had a beneficial effect by way of prevention within the last ten or twelve years; so may the regulations and care of the different boards of health, which, to a certain degree, should never be intermitted. Still I am of opinion, that the heat, not of a few days or weeks, but the mean heat of the summer season, is the grand governing cause, under Providence, that excites or depresses this alarming and dreadful scourge when it appears in our city.

---

An anonymous writer in the *Repository*, speaks of the beneficial effects he has witnessed from the use of *sub-borate of soda*, as an external application in cancer and scrofula. He relates three cases, which he considered to be cancer of the lip, that were perfectly cured by it; and one, in which the breast was the seat of the disease, respecting which, several surgeons "of the first respectability" had given it as their opinion, that the disease was too far advanced to admit of an operation with any probability of success, that was so much relieved by it, as to cause little uneasiness: the patient died of pneumonia six years afterwards. The sub-borate of soda was used in the proportion of half an ounce to a pint of water, occasionally adding two or three drachms of the extract of hyosciamus. The writer very properly speaks with much caution respecting the advantages which may be expected from the use of it in the generality of cases; he merely proposes it as "the most efficient topical application with which he is acquainted." That it may prove useful in some ill-conditioned ulcers is probable; and in those of cancer, where the disease is beyond the reach of our hitherto most efficient modes of treatment, it may be employed without blame; but we think it should not interfere with the means from which we should expect advantage in cases that may be considered as remediable.

*Case of a Child, aged six months, who swallowed a double-bladed Knife without Injury.*

Few subjects are more interesting than the contemplation of the wonderful manner in which the human frame accommodates itself to the various violences to which it is subject: compression upon the brain; the effusion of fluids into the pericardium, thorax, and abdomen; a musket-ball, or other extraneous body, in the midst of muscle, &c. all may remain a considerable length of time, without necessarily proving destructive. The human stomach is daily exposed to severe trials by the glutton and the drunkard, and daily it evinces its power of contending against such attacks, although it ultimately falls a sacrifice to their repetition or continuance.

If we are surprised at the efforts it is capable of in such instances, how much more must we wonder at those remarkable powers of adaptation by which it is sometimes enabled to remain uninjured, when such substances as nails, pins, knives, &c. are swallowed by accident.

The painful and ridiculous feat of the Indian jugglers in passing a blunt piece of iron, under the name of a sword, into their stomach, which certainly contributes to render them short-lived; and the instances we have of men actually swallowing knives to the number of twelve or thirteen, for a reward of spirits or wine; do not come within the intention of these observations: they are meant chiefly to apply to those cases where foreign substances have been inadvertently swallowed.

In the Transactions of the Royal Society, cases are recorded of knives being swallowed by adults, which forced their way through the coats of the stomach by producing inflammation, &c. or were removed by incision. We have also many histories of nails, padlocks, knives, &c. being swallowed without producing fatal consequences; but I am not aware of any case being recorded, where a knife remained so long in the stomach of so young a child, as in that of which I now give the particulars, and which, on that account, deserves to be preserved, if it has not already been communicated by the very respectable persons who, with myself, were witnesses to the facts.

Case.—March 16th, 1802. A child of Jonathan White's, Southgate, Chichester, about six months old, had a small double-bladed knife, about two inches and a half in length, given it to play with. On the return of its mother to the room, she sought in vain for the knife in all parts of the cradle in which the infant was lying: the child expressed some uneasiness at the stomach, from which the mother concluded it had swallowed the knife; the bowels were kept lax by the use of castor-oil, and the fæces soon began to grow black. The child took no food but milk; seemed often very uneasy in its stomach, and had slight febrile indisposition; yet it continued to look well, and was sufficiently fat.

May 24th.—The shortest blade was discharged by the bowels; the back of it very much corroded, its edges being ragged, uneven, and saw-like; the rivet was entirely dissolved. The general state of the child's health, as stated above.

June 10th.—The child, after being for a day or two more than usually uneasy, and rejecting every thing offered as food, brought from its stomach, in vomiting, one side of the horn handle, about two inches in length, very much softened, and bent double: a small bit of iron was passed a few days afterwards by stool. He frequently expresses great pain in his stomach and bowels, and starts much when asleep; has retained no nourishment for three days, and now looks much emaciated.

July 8th.—The child more emaciated, takes little food, and, unless when quieted by a decoction of poppies, expresses more pain, continually writhing. Its bowels are lax, and the stools have a black appearance, and the abdomen exhibits externally a degree of inflammation. His pulse is soft and moderate while asleep; the skin feels rough; has voided nothing since the horn handle.

July 24th.—To-day he passed a bit of iron, which was about half an inch in length, of a wedge-like shape, much corroded, and full of holes, and appearing to have been the large blade.

August 11th.—The child has been in a convalescent state for the last fortnight, grows fatter, and looks much better; has been more quiet, although he has not slept much; the decoction of poppies has been omitted for some time past; the pulse full and strong; sucks more heartily, and now eats sopped bread three or four times a-day. Yesterday and to-day it has been more uneasy: about five o'clock in the evening, vomited up its milk, together with the back of the knife, two and a half inches in length, pointed, and corroded at one end; the other nearly perfect, and first presented itself at the mouth: soon after, it vomited the other side of the horn handle, softened, the edges uneven, and dissolved. The child was much exhausted by its efforts, and soon fell asleep. The stools are some days of their natural colour, and sometimes black.

Dec. 20th.—The child is now in perfect health, remarkably robust, and has not experienced a day's illness since August.

The notes from whence I have taken the above particulars were made at the moment by Mr. J. N. Shelley, now a surgeon in the army, who was at that period my senior, and whose observations I can corroborate most fully.

W. H. BANKS, *Surgeon, Royal Navy.*

*Ryde, Isle of Wight; May 8th*, 1818.

*Journal of Science and the Arts.*

---

### *On the increasing Populousness of England.*

The inquiry instituted, and census taken, in 1801 and 1811, presented results as extraordinary as unexpected; showing an accelerated progress of increasing population in Great Britain at the close of the last century and beginning of the present, which was thought very unlikely to continue with like rapidity in future.

The proportion of births to deaths had been estimated at 11 to 10, about the middle of the past century;* and that estimate has not been deemed materially defective. In the latter part of the century (taking a period of twenty years) the proportion of registered baptisms to burials in all England and Wales, was found to be 13 to 10; and, on an average of the last five years of it, 137 to 100.† In the first decade of the present age, the proportion exhibited by the returns of the parish-abstracts was 148 to 100; and for the last five years of this decennial period, 151 to 100.‡

As the registers of baptisms are known to be more defective than those of funerals; among other reasons, because many dissenters from the established church bury their dead in the parish-cemetery, who have not their children baptised according to the rites of the church; and because private baptisms are excluded from some of the registers,§ and the interment of still-born and unbaptised children is in others included;|| it followed that the excess of births above deaths was still greater than the abstracts of parish-registers exhibited. Admitting a requisite, but conjectural, correction upon this ground, the proportion of births to deaths, on a medium of the ten first years of the present century, has been taken at 16 to 10;¶ a proportion considered to be quite extraordinary for a rich and well-peopled territory; showing a rate of increase, which, as remarked concerning it, cannot be permanent, and which it would be unreasonable to expect should endure for any long continuance.**

There seems reason, however, to believe that the accelerated

---

* *Dr. Short. New Obs.* 22 and 24.

† In twenty years (1780—1800), 5,014,899 baptisms, and 3,840,455 burials; annual average, 250,745 to 192,023, or 131: 100.

In five last years (1796—1800), average 255,426 to 186,000, or 137 : 100.

‡ In ten years (1801—1810), 2,878,906 baptisms, 1,950,189 burials, or 148: 100.

In five last years (1806—1810), average 297,000 to 196,000, or 151 : 100.

§ *Pop. Abs. Prel. Obs.* 22.

|| In the Bills of Mortality for London, abortive and still-born children are included in the burials, to the number of about 600 annually; *Price, Rev. Paym.* In ten years (1801—1810), 5,437; *Milne, Ann.* In the five last years (1813—1817), 3,551; or, on an average, 710; *Bills Mort.* The whole number of still-born must be much greater, being in proportion of 5 to 100 born alive; *Dr. Clark.* The unbaptised are not fewer, for more die in the first fortnight than are still-born; *ib.* but not all unchristened.

¶ Add one-sixth to the registered baptisms, and one-twelfth to the registered burials; *Malthus on Pop.* ii.

** Malthus.

progress of increase, exhibited by the growing ratio of excess of births above deaths to the whole population, has yet received no check, and that the augmentation of the people is proceeding with a rapidity as great in the second as in the first decade of the century. As this is a point of much moment in connexion with many important considerations, the grounds of the opinion now stated will be given, and with as much brevity as the nature of the subject allows.

The Bills of Mortality of London, annually published, exhibit in the past century an excess of burials above baptisms progressively diminishing, until nearly equalized in the latter part of it: the average of the last five years showing the proportion of 98 baptisms to 100 funerals.* From the beginning of the present century, the registered baptisms have exceeded the burials; the ratio for the first five years being 108 to 100,† and for ten nearly the same; and the excess has increased in the present decade,—the ratio being for the elapsed portion of it, 115 to 100, and for the last three years, 119 to 100.‡

The Bills of Mortality are not supposed to be quite accurate. It appears from the parish abstracts, returned under the Population-Acts, that, in the last twenty years of the past century, the proportion of baptisms to burials was 92 to 100; but, according to the Bills, 94 to 100;§ and in the first ten years of the present century, 111 to 100; but according to the Bills, 108 to 100.‖ Presuming that the Bills of Mortality will not prove to be now more inaccurate, compared with the abstracts to be returned for a future census, than heretofore, there appears to be sufficient evidence that the excess of births above the deaths in the metropolis is in progress of increase. The town, then, is no longer a drain upon the country for maintaining the number of its inhabitants, which it upholds, and even augments.

Mary-le-bone, which is not included within the Bills of Mortality, is the most populous parish in Great Britain. The number of its inhabitants, which was 63,982 according to the enumeration

---

* In five years (1781—1795), bap. 86,316, bu. 94,403; av. 17,263 : 18,881.

Five years (1796—1800), bap. 93,544, bu. 95,659; av. 18,709 : 19,132.

† In five years (1801—1805), bap. 100,553, bu. 92,856; av. 20,111 : 18,571.

Ten years (1801—1810), bap 199,797, bu. 185,736.

‡ In seven years (1811—1817), bap. 152,871, bu. 133,287; av. 21,839 : 19,041.

Three years (1815—1817), bap. 71,124, bu. 59,844; av. 23,708 : 19,921.

§ Abstracts of Par. Reg. 1781—1800, bap. 394,309, bu. 422,404

Bills of Mortality - - bap. 366,191, bu. 389,491

‖ Abstracts of Par. Reg. 1801—1810, bap. 210,454, bu. 188,910
Bills of Mortality - - bap. 199,797, bu. 185,655

in 1801, and 75,624 according to that of 1811, is almost a twelfth part of the population of the metropolis, and 125th of that of England. It equals, or nearly does so, the aggregate of other parishes contiguous to London, and comprising a portion of the suburbs, though not comprehended in the Bills of Mortality.*

The registered baptisms in this parish nearly equalled the burials in the ten years from 1781 to 1790, and exceeded them in the next ten, 1791 to 1800; as also in the ten following, 1801 to 1810; the proportion being severally, 96 to 100, 110 to 100, and 108 to 100.† The excess of registered births above deaths is become yet greater, being, for the seven years which have since elapsed, 138 to 100; and for the three last, 157 to 100.‡

In the parish of Mary-le-bone, the burials of persons denominated foreigners amount to rather more than 165 annually; and, if these were excluded, the excess of births above deaths would appear to be yet greater.

One of the most populous parishes beyond the precincts of the metropolis is Hampstead. Being a resort of the sick on account of the reputed salubrity of the spot, many sojourners die, and are interred, there; and the funerals, according to the abstracts returned for the census, continued to exceed the baptisms to the latest period of those returns (1810). The population of the place was 4,343 in 1801, and 5.483 in 1811; but the funerals in the intermediate ten years were 1,377, and baptisms 1,124. An accession of inhabitants replaced the deficiency, and augmented the number in no less a ratio than as 5 to 4.

In the last five years, the baptisms in this parish have been 646, and burials 642, or in the proportion of 101 to 100 nearly; instead of the former ratio 82 to 100, on the medium of ten years.§

As an instance of a rural parish in the vicinity of the metropolis, more than eight and less than ten miles distant from it, the parish of Edgeware has been taken, and upon no other ground of selection besides the accidental circumstance of facility in consulting its register.

---

* Pancras, Paddington, Kensington, and Chelsea, contained 53,922 inhabitants in 1801, and 80,080 in 1811. To the five out-parishes mentioned, Camberwell should be added; it contained 7,059 persons in 1801, and 11,309 in 1811.

† In ten years, 1781—1790, bap. 12,325, bu. 12,871.
1791—1800, bap. 17,410, bu. 14,880.
1801—1810, bap. 18,991, bu. 17,553.
*Printed Acc. rec. and disb. of the rates of St. Mary-le-bone.*

‡ In seven years (1811—1817, bap. 57,432, bu. 12,660; av. 2,490 : 1,809.

Three years (1815—1817), bap. 7,977, bu. 5,089; av. 2,659: 1,696.

§ In ten years (1801—1810), bap. 1,124, bu. 1,377.
Five years (1813—1807), bap. 646, bu. 642; av. 129: 128.

The proportion of births to deaths has in this parish increased from the ratio of 123 : 100, which the average of ten years exhibited to that of 138 : 100, on the medium of the seven subsequent years; and 147 : 100 in the three last.*

Considering that Mary-le-bone, Hampstead, and Edgeware, are no unfair specimens of three classes of parishes in and near London, it is apparent from these instances, in concurrence with the Bills of Mortality, that, within the metropolis and its immediate vicinity, (the population of which is not less than a tenth of that of Great Britain,†) the number of inhabitants has continued to increase since the census of 1811, and at an accelerated rate. And, as the number of inhabitants of all Great Britain has hitherto been found to increase faster than that of the metropolis, it seems fairly to be inferred, as a probable result to be expected from the next census, that the population of all Great Britain will appear to have been increasing to this time with yet greater rapidity than the results of the former census showed.

To bring this conclusion to the test of a comparison with information collected from remote parts of the kingdom, would require more extensive research than can well be undertaken by an individual. The registers of a few distant parishes have been consulted; and the results, as might be expected, are various. It is, however, conceived that the continued rapid growth of the capital city does assuredly indicate a continuance of quick increase of populousness of the country in general.

---

* In ten years, 1801—1810, bap. 111, bu. 9.
Seven years, 1811—1817, bap. 105, bu. 76; av. 15 : 11.
Five years, 1813—1817, bap. 87, bu. 59; av. 17 : 12.
Population, 412 in 1801, and 543 in 1811.

† The population of London and its neighbourhood, within eight miles around the cathedral of St. Paul's, was 1,220,000, according to the census of 1811; and that of all Great Britain, with the army and navy, was 12,596,803. *Pop. Abs.* Carrying the vicinage to ten miles, the proportion is as stated.

*Journal of Science and the Arts.*

